# Secukinumab Mitigates Cisplatin-Induced Nephrotoxicity and Enhances Cisplatin Cytotoxicity in MCF-7 Cells via IL-17A/NF-κB Axis Modulation

**DOI:** 10.3390/toxics14050424

**Published:** 2026-05-12

**Authors:** Faiz N. Alenezi, Marwa S. Zaghloul, Manar A. Nader, Marwa E. Abdelmageed

**Affiliations:** 1 Department of Pharmacology and Toxicology, Faculty of Pharmacy, Mansoura University, Mansoura 35516, Egypt; faiz.mans.edu.eg@std.mans.edu.eg (F.N.A.); dr_marwasalah@mans.edu.eg (M.S.Z.); manarahna@mans.edu.eg (M.A.N.); 2 Department of Pharmacology and Toxicology, Faculty of Pharmacy, Mansoura National University, Gamasa 7731168, Egypt

**Keywords:** Cisplatin, Secukinumab, acute kidney injury, MCF-7 Cells, IL-17A/NF-κB/P38, autophagy, oxidative stress, apoptosis

## Abstract

Objective: The existing work was designed to appraise whether Secukinumab diminishes acute kidney injury in a Cisplatin- induced rat model and to explore the potential underlying mechanisms for this protective effect. Methods: In vivo study, rats were distributed haphazardly into five sets (six animals in each group): control, Secukinumab control, Cisplatin (8 mg/kg, a single dose, intraperitoneally (IP)), and two pretreated groups; Secukinumab (10 and 20 mg/kg single subcutaneous (SC) injection) + Cisplatin. Blood samples and kidney tissues were gathered and analyzed histopathologically and biochemically. In vitro investigation, MCF-7 human breast cancer cells were treated with Cisplatin alone with Secukinumab, and cell viability (MTT assay), combination index, and apoptosis-related markers were analyzed. Results: Secukinumab administration lowered serum levels of BUN, creatinine and LDH with marked elevation in renal TAC and a significant reduction in MDA, iNOS, KIM-1 and NGAL compared to Cisplatin. Additionally, Secukinumab pre-treatment markedly suppressed the inflammatory process and enhanced autophagy, reflected by elevated AMPKα1, SIRT1, and Beclin-1, accompanied by reduced P38 MAPK and NF-κB p65 (Phospho-Ser536) levels and expression levels of IL-6 and P62/SQSTM1 in kidney tissues, contrasted with the Cisplatin group. Secukinumab administration effectively protected against kidney injury, and histopathological examinations of the kidneys confirmed these results. On the other hand, in vitro study results revealed that the combination of Cisplatin and Secukinumab had a synergistic cytotoxic effect and an enhancing effect on the apoptotic pathway (increased P53 and BAX and decreased BCL-2). Secukinumab effectively protects against Cisplatin- induced acute kidney injury by decreasing oxidative stress, inflammation, and enhancing autophagy. Additionally, it synergizes with Cisplatin in vitro to promote cancer cell apoptosis, highlighting its dual reno-protective and anticancer potential.

## 1. Introduction

The kidney is a vital metabolic organ that maintains the stability of the internal environment and controls the balance of water and electrolytes. It also performs an endocrine role by secreting erythropoietin, which helps prevent anemia [1,2]. Chemotherapeutic medications, antibiotics, infection, or surgery can result in acute kidney injury (AKI) [3]. One frequent chemotherapeutic medication used in clinics is Cisplatin [4]. It can induce AKI even if it has therapeutic effects on several malignancies. As a result, Cisplatin is frequently employed in animal research to create kidney damage models [5].

Cisplatin’s nephrotoxicity is intimately linked to oxidative stress, inflammatory responses, and apoptosis [6]. Effective prevention strategies are needed to mitigate Cisplatin-induced AKI, which may include medication therapy. In addition to causing oxidative stress and apoptosis, Cisplatin triggers inflammatory reactions in renal tissue by activating nuclear factor kappa B (NF-κB), the master regulator of inflammation, that drives the expression of pro-inflammatory cytokines and chemokines, thereby amplifying nephrotoxicity [7,8].

Recent evidence highlights a significant role for interleukin-17A (IL-17A) in augmenting NF-κB-mediated inflammatory signaling in cisplatin-induced acute kidney injury (AKI). IL-17A, a pro-inflammatory cytokine produced by Th17 cells, has been shown to enhance NF-κB activation and promote leukocyte recruitment and cytokine release in experimental models of renal inflammation [9,10,11]. Elevated IL-17A levels can thereby exacerbate cisplatin-induced renal damage by sustaining NF-κB activity, leading to increased expression of TNF-α, IL-6 and other mediators that contribute to tubular cell injury.

Important signaling molecules, such as the histone deacetylase Sirtuin 1 (SIRT1), have also been linked to Cisplatin-induced nephrotoxicity [12]. SIRT1 plays a key role in regulating metabolic responses to nutrient availability [13]. In addition, SIRT1 suppresses apoptosis, inflammation, and oxidative stress by deacetylating several substrates, including the p65 subunit of NF-κB [14,15]. Therefore, SIRT1 has emerged as a promising therapeutic target for the treatment of various inflammatory disorders. Notably, overexpression of SIRT1 in renal tubular cells has been shown to alleviate Cisplatin-provoked AKI by reducing apoptotic cell death and oxidative stress [16], partially through downregulation of NF-κB-dependent inflammatory signaling [12,17]. Therefore, highlighting these molecules as possible targets for reducing Cisplatin- induced nephrotoxicity.

Collectively, these mechanistic insights suggest a network in which IL-17A potentiates NF κB-driven inflammation, intensifying Cisplatin-induced renal damage, while SIRT1 mitigates these effects through negative regulation of NF κB signaling. This conceptual framework supports the investigation of therapies that target both inflammatory and stress regulatory pathways to ameliorate Cisplatin-associated kidney injury.

Secukinumab is a human monoclonal antibody that specifically targets and prevents interleukin (IL)-17A from interacting with the IL-17 receptor. Patients with psoriasis and ankylosing spondylitis benefit from it for a long time [18,19]. Additionally, Secukinumab has been shown to protect rats with severe sepsis by inhibiting the IKBα/NF-κB inflammatory signaling pathway [20]. Recent findings have also indicated that in the renal tissue of rats given sunitinib, Secukinumab considerably reduced NLRP3 inflammasome-mediated inflammation while increasing Beclin-1-mediated autophagy, cleaved caspase-3 expression, and interstitial fibrosis [21].

Secukinumab, a fully human monoclonal antibody targeting IL-17A, has demonstrated a favorable safety profile in both clinical and preclinical studies. Reported adverse effects are generally mild to moderate and include an increased susceptibility to infections, particularly upper respiratory tract infections, as well as occasional neutropenia. Notably, IL-17 inhibition has been associated with exacerbation of inflammatory bowel disease in certain patients [22,23,24,25,26].

Cytokines and chemokines are key regulators of the immune system and play a central role in the pathogenesis of various kidney diseases [27]. Although the effectiveness of IL-17A antagonists in inflammatory disorders has been well documented, their role in cancer- and chemotherapy-related kidney diseases remains insufficiently investigated. Collectively, these findings may repurpose Secukinumab as a candidate therapy to alleviate the renal toxicity of Cisplatin and to rescue the renal functionality. In the current work, we focus on investigating the potential protective action of Secukinumab in a rat model of Cisplatin-provoked nephrotoxicity. Additionally, we evaluated whether Secukinumab interferes with or enhances the anticancer efficacy of Cisplatin in human breast cancer cells (MCF-7), to ensure that its nephroprotective action does not compromise the chemotherapeutic potential of Cisplatin. The study aimed to demonstrate a “dual benefit” profile: protecting a vital organ (the kidney) in vivo while maintaining or enhancing the therapeutic strike against breast cancer cells in vitro. This comprehensive approach is vital for proposing any potential adjuvant therapy in clinical oncology.

## 2. Methods and Materials

### 2.1. Materials

Cisplatin was purchased as an injectable solution (Cisplatine^®^ Mylan (1 mg/mL), Oncotec Pharma production, Dessau-Roblau, Germany) and Secukinumab was acquired as a commercially available prefilled pen containing 150 mg/mL of the drug (COSENTYX^®^, Novartis Pharmaceuticals Corporation, East Hanover, NJ, USA) and was diluted in (0.9%) normal saline. Other chemicals employed were of superior analytical quality.

### 2.2. In Vivo Study

#### 2.2.1. Animals

Adult male Wistar rats (12–14 weeks old, weighing 200–250 g) were obtained from VACSERA, Giza, Egypt, and housed four per cage with free access to food and water. Animals were maintained under controlled conditions (25 ± 2 °C, 40–70% humidity, 12/12 h light/dark cycle) in an air-conditioned facility. All procedures were approved by the Animal Care and Use Committee, Mansoura University (Approval code No. MU-ACUC (PHARM.PhD.23.08.27)) and conducted following NIH guidelines for laboratory animal care (NIH publication no. 85-23, revised 2011).

#### 2.2.2. Experimental Design

**Figure 1:**
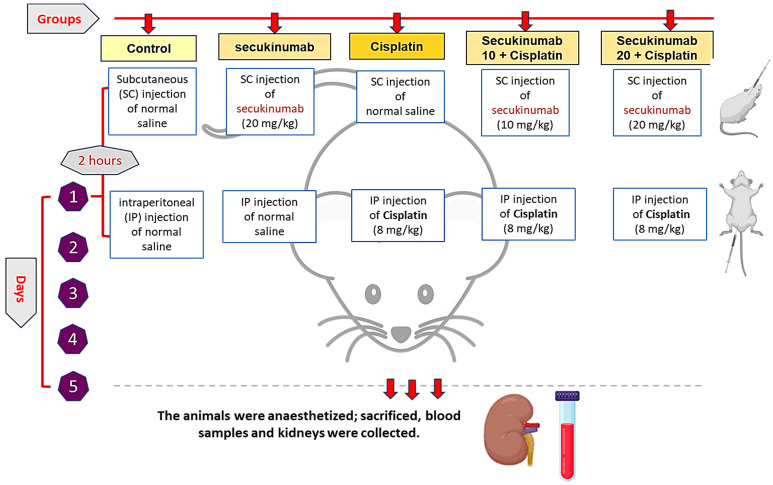
Schematic representation of the experimental design.

After 10 days of acclimatization, male Wistar rats were then randomly assigned to five groups (*n* = 6) (Figure 1):

Group I (Control group): normal saline injected subcutaneously (SC), and after 2 h injected IP.

Group II (Secukinumab group): 20 mg/kg of Secukinumab given SC and a single intraperitoneal (IP) injection of normal saline after 2 h.

Group III (Cisplatin group): SC injection of normal saline and IP with Cisplatin (8 mg/kg) after 2 h [28,29,30,31].

Group IV (Secukinumab 10 + Cisplatin group): injection with 10 mg/kg of Secukinumab SC, then 2 h later, Cisplatin was administered IP.

Group V (Secukinumab 20 + Cisplatin group): injection with 20 mg/kg of Secukinumab SC, then after 2 h. Cisplatin was administered IP.

The 20 mg/kg Secukinumab-only group served as a comprehensive safety control for both treated groups, adhering to ethical guidelines for animal welfare [32].

The doses of Secukinumab were selected based on an initial pilot study. Our pilot study included 3 doses of Secukinumab (5, 10 and 20 mg/kg) administered with Cisplatin on serum biomarkers, which demonstrated that the dose (5 mg/kg) was not effective upon assessment of the general parameters, so we excluded this dose and continued our experiment with doses (10 and 20 mg/kg) and these doses were consistent with findings from previous study [20].

Rats were anesthetized with thiopental sodium (50 mg/kg, IP) 3 days post-Cisplatin injection, blood was collected via retro-orbital puncture, allowed to clot for 30 min, and centrifuged at 3000× *g* for 15 min at 4 °C, and serum was collected. Rats were then sacrificed, and kidneys were harvested, rinsed with PBS, and blotted dry. Right kidneys were frozen at –80 °C in PBS (10% *w*/*v*) and later homogenized; left kidneys were fixed in 10% formalin.

#### 2.2.3. Investigations of Kidney Function Biomarkers in Serum

Serum creatinine and blood urea nitrogen (BUN) (Cat. No. MD1001111 and TK41041, respectively) were assessed using commercial kits obtained from Spinreact, Girona, Spain. All procedures were conducted according to the manufacturer’s instructions. Additionally, lactate dehydrogenase (LDH) levels in serum were measured using a kit (Agappe, Kerala, India, Cat. No. 11407001) following the manufacturer’s instructions.

#### 2.2.4. Histopathological Investigations

The fixed kidney tissues were embedded in paraffin, sectioned at 4–5 μm, and stained with hematoxylin and eosin (H&E) and examined under a light microscope at 100× magnification. The pathologist was blinded to the study groups. Pathological changes were independently assessed using a semiquantitative grading scale [33]. Semiquantitative assessment of kidney alterations was examined through histological categorization as summarized in Table 1. The assessments were performed using microscopic examination (Leica Imaging Systems, Cambridge, UK).

#### 2.2.5. Estimation of Oxidative Stress Markers

Renal tissue samples were analyzed for malondialdehyde (MDA) and total antioxidant capacity (TAC) levels according to the manufacturer’s instructions (Biodiagnostic kits, Cairo, Egypt, Cat. No. MD2529 and TA2513, respectively).

#### 2.2.6. ELISA Assessment

The renal levels of kidney injury molecule 1 (KIM-1) and neutrophil gelatinase-associated lipocalin (NGAL) were determined using commercial ELISA kits according to the manufacturer’s instructions (KIM-1; Cat no: CSB-E08808r and NGAL; Cat no: CSB-E09409r, Cusabio, Houston, TX 77054, USA), respectively.

Additionally, the levels of nuclear factor kappa B (NF-κB p65 (Phospho-Ser536)), rat Nad-dependent protein deacetylase sirtuin-1 (SIRT1) and P38 mitogen-activated protein kinase (P38 MAPK) were determined in kidney tissue homogenates using commercial ELISA kits according to the manufacturer’s instructions (NF-κB p65 (Phospho-Ser536); Cat no: MBS9511033, MyBioSource, San Diego, CA, USA), (SIRT1; Cat no: E1145Ra, BT lab, Shanghai, China), (P38 MAPK; Cat no: RTFI00943, Assay Genie, Dublin 2, Ireland).

Furthermore, inducible nitric oxide synthase (iNOS), AMP-activated protein kinase α1 (AMPKα1) and Beclin-1 were determined in kidney tissue homogenates using commercial ELISA kits according to the manufacturer’s instructions (iNOS; Cat no: RTFI00088, Assay Genie, Dublin 2, Ireland), (AMPKα1; Cat no: DYC3197-2, R&D systems, Minneapolis, MN 55413, USA), (Beclin-1; Cat no: CSB EL002658RA, Cusabio, Houston, TX 77054, USA).

#### 2.2.7. Immunohistochemical Analysis

Polyclonal antibodies were obtained from (Thermo-Fisher Scientific Anatomical Pathology, Waltham, MA, USA, Cat no. PA5–27617 for IL-6 and Cat No. PA5-20839 for P62/SQSTM1, respectively) for use in the Biotin–Avidin complex process [35] and for immunohistochemical analysis of IL-6 and P62/SQSTM1 in kidney tissues. Slides were analyzed using an MBL4000-T-F-LED microscope. For each group, three sections were assessed, and the percentage of positive areas was quantified using ImageJ software version 1.54 (FIJI, National Institutes of Health, Bethesda, MD, USA).

### 2.3. In Vitro Experiment

#### 2.3.1. Cell Culture

The human breast adenocarcinoma cell line MCF-7 was cultured in Dulbecco’s Modified Eagle Medium (DMEM) supplemented with 10% fetal bovine serum and 1% penicillin–streptomycin at 37 °C in a humidified 5% CO_2_ atmosphere.

#### 2.3.2. Cell Seeding, Experimental Design and Drug Treatment

MCF-7 cells were settled in 96-well plates (5 × 10^3^ cells/well) 24 h before treatment, then allocated to the following groups for 48 h: Control group; seeded with vehicle, Secukinumab group: treated with increasing concentrations of Secukinumab (0.1–50 µM). Cisplatin group: treated with increasing concentrations of Cisplatin (0.5 to 40 µM). Combination group: managed with Cisplatin with a fixed, non-toxic concentration of Secukinumab (selected based on preliminary viability experiments), this group was used to calculate the combination index (CI) to permit the correct estimation of pharmacological interactions between Cisplatin and Secukinumab. Chou–Talalay CI method was utilized (CompuSyn software program, Combosyn Inc., Bergen County, NJ, USA) to estimate the CI [36]. CI values were interpreted as follows: CI < 1 indicates synergism, CI = 1 indicates an additive effect, and CI > 1 indicates antagonism. Further classification of synergism was defined as follows: CI = 0.3–0.7 (strong synergism), 0.7–0.9 (moderate synergism), and 0.9–1.1 (nearly additive) [36,37].

#### 2.3.3. Cell Viability Analysis

Cell viability was assessed using the MTT assay. After treatment, 20 µL of MTT solution (5 mg/mL) was added to each well and incubated for 4 h at 37 °C. The resultant formazan crystals were dissolved in DMSO, and absorbance was measured at 570 nm. Cell viability was assessed via the subsequent formula:

$$\mathrm{Cell}\;\mathrm{viability}\;(\%)=\left(\frac{\mathrm{Absorbance}\;\mathrm{of}\;\mathrm{treated}\;\mathrm{cells}}{\mathrm{Absorbance}\;\mathrm{of}\;\mathrm{control}\;\mathrm{cells}}\right)\times 100$$

#### 2.3.4. Determination of Half-Maximal Inhibitory Concentration (IC_50_) and CI

IC_50_ values for Cisplatin alone and in combination with Secukinumab were calculated from dose–response curves.

### 2.4. Statistical Analysis

The Shapiro–Wilk test was utilized for normality assessment. Parametric results were evaluated by one-way ANOVA with Tukey–Kramer’s post hoc test and are presented as mean ± SD, while nonparametric ones were evaluated via Kruskal–Wallis as median ± interquartile range. Statistical investigations were performed using SPSS 25.0 (IBM/SPSS, Chicago, IL 60606, USA), with *p* < 0.05 considered significant. The software GraphPad Prism version 8.0.1 was utilized to examine the in vitro results.

## 3. Results

### 3.1. In Vivo Experiment Results:

#### 3.1.1. The Impact of Secukinumab (10 & 20 mg/kg) on Cisplatin-Induced Alterations in Serum Kidney Function Biomarkers and LDH

Table 2 demonstrated that Cisplatin injection significantly affected functional markers (creatinine (F = 37.047, *p* < 0.001) and BUN (F = 116.134, *p* < 0.001)) and tissue damage biomarker (LDH) (F = 11.349, *p* < 0.001) compared to the control group. Conversely, pre-treatment of Secukinumab 20 mg/kg markedly reduced these levels in contrast to the Cisplatin group.

Only the BUN level was significantly reduced following treatment with Secukinumab 10 mg/kg.

#### 3.1.2. The Impact of Secukinumab (10 and 20 mg/kg) on Cisplatin-Induced Kidney Structure Alterations

Control and Secukinumab control groups showed a normal architecture of glomeruli and renal tubules, in contrast to the Cisplatin group, which showed diffuse tubular degeneration with abundant intraluminal hyaline cast with scattered multifocal aggregates of lymphocytes and RBCs.

Meanwhile, the Secukinumab (10 mg/kg) + Cisplatin group showed tubular degeneration and necrosis with dense interstitial fibrosis mixed with a high number of inflammatory cells.

**Figure 2:**
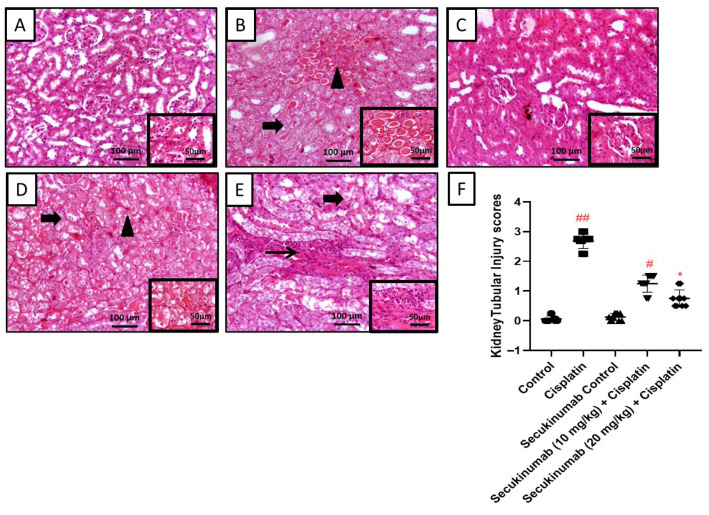
The impact of Secukinumab (10 & 20 mg/kg) on Cisplatin-induced kidney structure alterations. (**A**) The control group showed a normal architecture of the glomerulus and renal tubules. (**B**) The Cisplatin group showed diffuse tubular degeneration with abundant intraluminal hyaline cast with scattered multifocal aggregates of lymphocytes and red blood cells (RBCs). (**C**) The Secukinumab control group showed a normal architecture of glomeruli and renal tubules. (**D**) The Secukinumab (10 mg/kg) + Cisplatin group showed tubular degeneration and necrosis with dense interstitial fibrosis and inflammatory cells. (**E**) The Secukinumab (20 mg/kg) + Cisplatin group showed moderate tubular degeneration with intraluminal sloughed necrotic cells, interstitial congestion, inset. Image magnification = 100×, inset = 400×. (**F**) Scatter dot plots displaying kidney tubular injury scores. Thin arrow: fibrosis + inflammation; thick arrow: tubular swelling and necrosis; arrowhead: hyaline degeneration or intraluminal desquamated epithelium (cellular cast); circle: tubular dilation. Data were expressed as median ± interquartile range (IQR), *n* = 6 and were statistically analyzed using Kruskal–Wallis test followed by Dunn multiple comparisons test. #, ## indicate significant and highly significant differences compared with the control group, respectively; * indicates a significant difference compared with the Cisplatin group.

While Secukinumab (20 mg/kg) + Cisplatin showed moderate tubular degeneration with intraluminal sloughed necrotic cells, interstitial congestion and desquamation of tubular epithelial cells (Figure 2).

Secukinumab appears to exhibit superior efficacy at the higher dose (20 mg/kg), as evidenced by more significant improvements in the assessed biochemical and histopathological parameters. Therefore, the Secukinumab (20 mg/kg) dose was employed in the subsequent pathway and mechanistic analyses to further elucidate its molecular mode of action.

#### 3.1.3. The Impact of Secukinumab (20 mg/kg) on Cisplatin-Induced Alterations in Early Biomarkers for Acute Kidney Injury: KIM-1 and NGAL in Kidney Tissue

**Figure 3:**
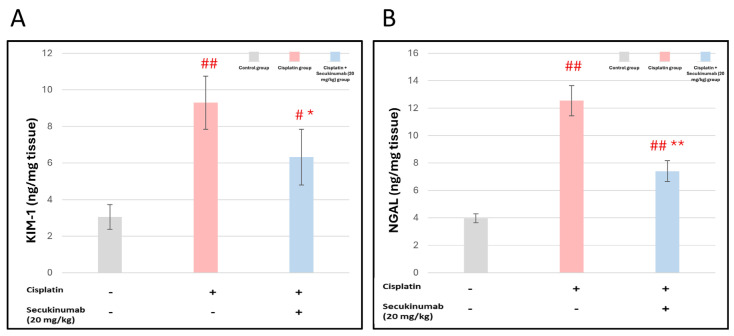
The impact of Secukinumab (20 mg/kg) on Cisplatin-mediated alterations in early biomarkers for acute kidney injury: (**A**) KIM-1 and (**B**) NGAL in kidney tissue. Data are presented as mean ± SD (*n* = 5). Statistical evaluation was carried out using one-way ANOVA, followed by the Tukey–Kramer post hoc test. #, ## significant and highly significant differences matched to the control group, respectively, and *, ** represents significant and highly significant differences matched to the Cisplatin group, respectively. KIM-1: Kidney Injury Molecule-1, NGAL: Neutrophil Gelatinase-Associated Lipocalin.

Renal damage provoked by Cisplatin was reflected by a significant rise in KIM-1 (F = 29.779, *p* < 0.001) and NGAL (F = 146.478, *p* < 0.001) in kidney tissue matched to the control group. Pre-treatment with Secukinumab (20 mg/kg) markedly mitigated these elevations compared to the Cisplatin group (Figure 3A,B).

#### 3.1.4. The Impact of Secukinumab (20 mg/kg) on Cisplatin-Induced Alterations in Oxidants and Antioxidant Biomarkers in Kidney Tissues

**Figure 4:**
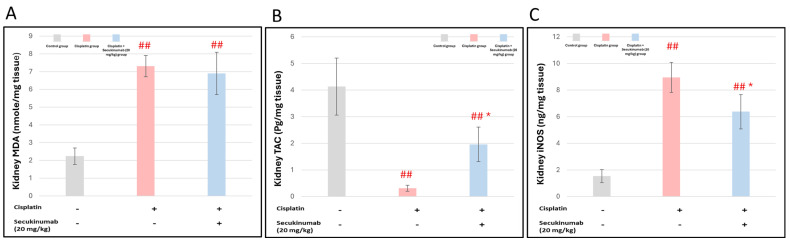
The impact of Secukinumab (20 mg/kg) on Cisplatin-induced alterations in oxidants and antioxidant biomarkers in kidney tissues. (**A**) MDA, (**B**) iNOS, (**C**) TAC. Data are presented as mean ± SD (*n* = 5). Statistical evaluation was carried out using one-way ANOVA, followed by the Tukey–Kramer post hoc test. ## highly significant difference compared with the control group, * significant difference compared with the Cisplatin group. MDA: Malondialdehyde; TAC: Total antioxidant Capacity; iNOS: Inducible nitric oxide synthase.

Cisplatin injection to rats significantly induced oxidative stress, as evidenced by enhanced lipid peroxidation as indicated by the elevated MDA (F = 59.554, *p* < 0.001) level, increased iNOS activity (F = 66.678, *p* < 0.001), and diminished TAC (F = 34.888, *p* < 0.001) in kidney tissues compared to the control group. On the contrary, Secukinumab (20 mg/kg) pre-treatment significantly improved these effects (Figure 4A–C).

#### 3.1.5. The Impact of Secukinumab (20 mg/kg) on Cisplatin-Induced Alterations in Stress Response-Inflammation-Autophagy Molecular Pathways

**Figure 5:**
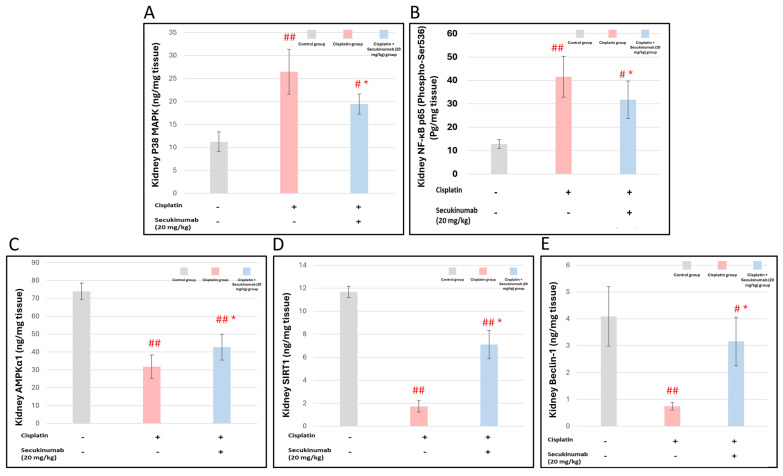
The impact of Secukinumab (20 mg/kg) on Cisplatin-induced alterations in stress response-inflammation-autophagy molecular pathways. (**A**) P38 MAPK, (**B**) NF-κB p65 (Phospho-Ser536) (**C**) AMPKα1, (**D**) SIRT1, and (**E**) Beclin-1. Data are presented as mean ± SD (*n* = 5). Statistical evaluation was carried out using one-way ANOVA, followed by the Tukey–Kramer post hoc test. #, ## significant and highly significant differences matched to the control group, respectively, * a significant difference matched to the Cisplatin group. MAPK: mitogen-activated protein kinase; NF-κB: Nuclear Factor Kappa B Subunit; SIRT1: Sirtuin 1; AMPK: AMP-activated protein kinase.

**Figure 6:**
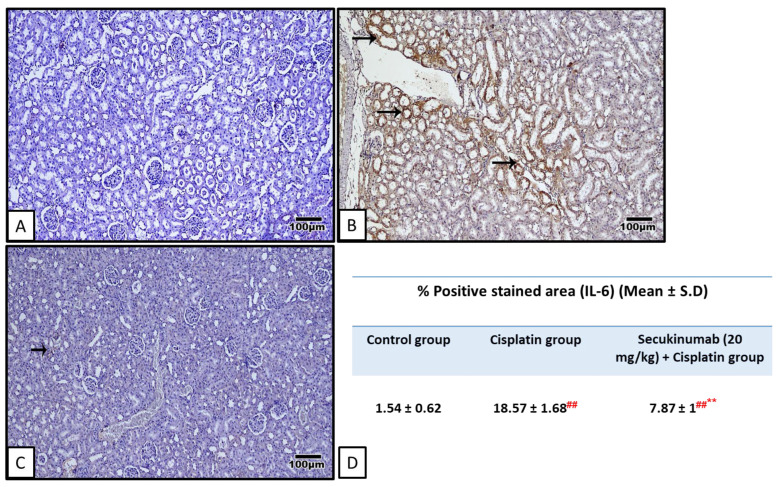
The impact of Secukinumab (20 mg/kg) on Cisplatin-mediated alterations in kidney expression level of interleukin-6 (IL-6). (**A**) The control group showed negative IL-6 immunostaining. (**B**) The Cisplatin group showed strong IL6 immunostaining (arrows) in the renal tubules. (**C**) The Secukinumab (20 mg/kg) + Cisplatin group showed weak IL-6 immunostaining (arrow). Image magnification = 400×. (**D**) The table represents the % positive-stained areas in different groups. Brown staining indicates positive immunoexpression, and blue staining represents hematoxylin counterstaining. Data are expressed as mean ± SD. Statistical analysis was performed using one-way ANOVA followed by Tukey–Kramer post hoc test. ##, ** highly significant difference in relation to control and Cisplatin groups, respectively.

**Figure 7:**
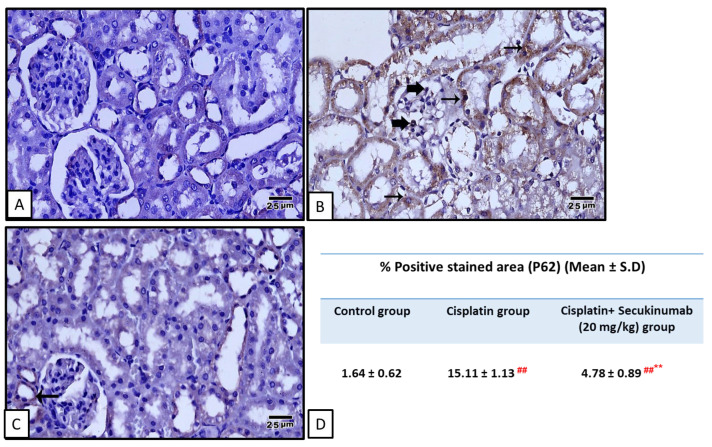
The impact of Secukinumab (20 mg/kg) on Cisplatin-mediated alterations in kidney expression level of P62/SQSTM1. (**A**) The control group showed negative P62/SQSTM1 immunostaining in glomerular cells or renal tubules. (**B**) The Cisplatin group showed strong P62/SQSTM1 immunostaining (thick arrows) in glomerular cells and in tubular cells (thin arrows). (**C**) The Secukinumab (20 mg/kg) + Cisplatin group showed weak P62/SQSTM1 immunostaining (thin arrow) in tubular cells and absence in glomerular cells. Image magnification = 400×. (**D**) The table represents the % positive-stained areas in different groups. Brown staining indicates positive immunoexpression, and blue staining represents hematoxylin counterstaining. Data are expressed as mean ± SD. Statistical analysis was performed using one-way ANOVA followed by Tukey–Kramer post hoc test. ##, ** highly significant difference in relation to control and Cisplatin groups, respectively.

Injection of Cisplatin significantly increased P38 MAPK (F = 26.264, *p* < 0.001) and NF-κB p65 (Phospho-Ser536) (F = 22.301, *p* < 0.001) (Figure 5A,B), and immuno- expression of IL-6 (F = 272.619, *p* < 0.001) (Figure 6) and sequestosome-1 (P62/SQSTM1) (F = 110.240, *p* < 0.001) (Figure 7) concomitant with a significant decrease in AMPKα1, SIRT1, and Beclin-1 levels (Figure 5C–E) in kidney tissues versus controls while Secukinumab pre-treatment reduced these markers and increased AMPKα1 (F = 62.030, *p* < 0.001), SIRT1 (F = 48.818, *p* < 0.001) and Beclin-1 (F = 21.901, *p* < 0.001) levels in kidney tissues compared with the Cisplatin group.

### 3.2. In Vitro Study Results

#### Effect of Secukinumab on MCF-7 Cell Viability

**Figure 8:**
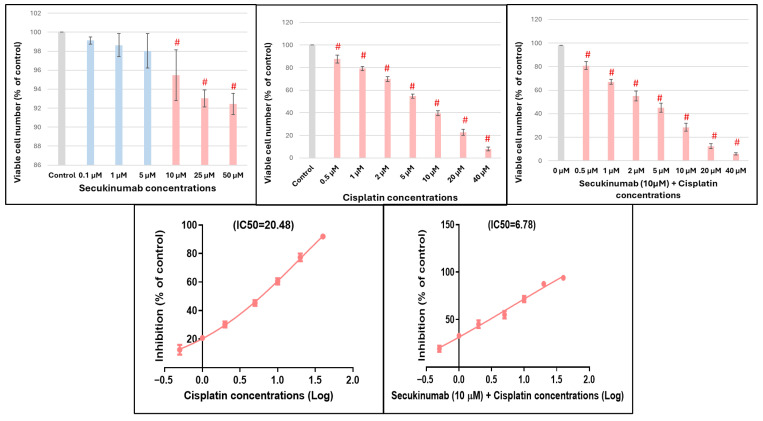
Effect of Cisplatin, Secukinumab and their combination on MCF-7 cell viability. Data are expressed as % control and presented as mean ± SD. Statistical analysis was performed using one-way ANOVA followed by Tukey–Kramer post hoc test. # significant difference in relation to control group.

As shown in Figure 8, Secukinumab alone did not significantly affect cell viability across all tested concentrations. Cell viability remained above 90% compared to the control group, indicating that Secukinumab is non-cytotoxic to MCF-7 cells within the tested concentration range. A concentration-dependent reduction in cell viability was observed by Cisplatin (0.5–40 µM (F = 992.5, *p* < 0.0001)). The IC_50_ value of Cisplatin alone was approximately 12 μM, confirming its cytotoxic effect on MCF-7 cells.

As illustrated in Figure 8, co-treatment significantly enhanced Cisplatin-induced cytotoxicity compared to Cisplatin alone (F = 633.3, *p* < 0.0001). The IC_50_ value of Cisplatin was markedly reduced from approximately 12 µM to 5 µM in the presence of Secukinumab, indicating enhanced sensitivity of MCF-7 cells to Cisplatin. IC_50_ values showed a significant decrease in Cisplatin concentration required to achieve 50% growth inhibition when combined with Secukinumab. This reduction indicates synergistic enhancement of Cisplatin cytotoxicity rather than an additive or antagonistic interaction.

CI values were consistently below 1 across all tested concentrations, ranging from 0.88 at low Cisplatin doses to 0.48 at higher concentrations, indicating strong to very strong synergism (Table 3).

**Figure 9:**
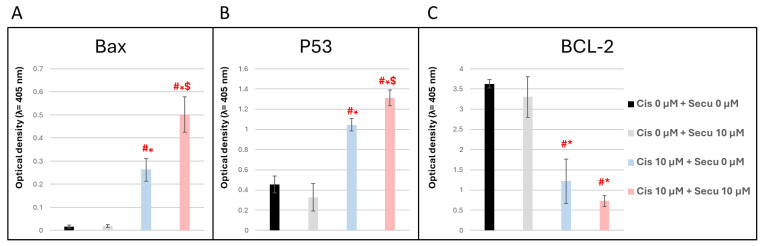
Consequence of combined Secukinumab and Cisplatin on the apoptotic effect of Cisplatin in MCF-7 cells. (**A**) BAX, (**B**) P53, (**C**) BCL-2. Data are expressed as mean ± SD. Statistical analysis was performed using one-way ANOVA followed by Tukey–Kramer post hoc test. # * $ Significant difference in relation to control group (Cis 0 µM + Secu 0 µM), Secukinumab-only treatment (cis 0 µM + Secu 10 µM) and Cisplatin-only treatment (cis 10 µM + Secu 0 µM), respectively. BAX: BCL-2-associated X protein; P53: tumor protein P53; BCL-2: B-cell lymphoma 2.

ELISA results from the current study indicated that the combined treatment with Secukinumab and Cisplatin significantly increased P53 (F = 95.78, *p* < 0.0001) and BAX (F = 128.5, *p* < 0.0001) expression compared to treatment with either drug alone. Additionally, the combination therapy resulted in a more pronounced reduction in BCL-2 (F = 57.08, *p* < 0.0001) levels than when the agents were used individually (Figure 9A–C). These results suggest that using Secukinumab in combination with Cisplatin markedly enhances antitumor efficacy while minimizing drug-related side effects.

## 4. Discussion

The present investigation displayed that nullification of IL-17A with Secukinumab improved cisplatin-induced AKI at functional, molecular, and histopathological levels. Mechanistically, our data revealed that Secukinumab reduced NF-κB– and p38 MAPK–driven inflammatory signaling downstream of IL-17A and IL-6.

In our experimental model of DEXA-induced NASH in rats, no overt signs of toxicity were observed upon Secukinumab administration, as evidenced by the absence of mortality, behavioral abnormalities, or significant deterioration in biochemical parameters beyond those induced by DEXA itself [22,23,24,25,26]. On the other hand, acute toxicity testing according to OECD 423 guidelines was not performed, as Secukinumab is a biologic agent with an established safety profile, and such protocols are primarily intended for small chemical entities rather than monoclonal antibodies [22,23,24,26,38].

Secukinumab also reduced nitrosative/oxidative stress; iNOS and MDA, whereas it restored antioxidant capacity, TAC, and restored SIRT1/AMPKα1/autophagy crosstalk, Beclin-1/P62/SQSTM1, toward adaptive cytoprotecting rather than injury.

These changes are associated with enhanced renal function (serum creatinine, BUN) and reduced tubular injury biomarker levels (KIM-1, NGAL), mutually with recovered histologic alterations and reduced IL-6/P62/SQSTM1 expression. The integrated picture aligns with current paradigms of Cisplatin-AKI pathogenesis that comprise oxidative stress, apoptosis/regulated necrosis, inflammation, and autophagy remodeling as dominant drivers of tubular injury and repair [6,39].

Our data revealed that the reductions in serum creatinine and BUN in Secukinumab-treated rats displayed rescue of glomerular filtration rate and tubular function. Notably, the associated decreases in KIM-1 and NGAL reinforce tubular-centric protection, as they are confirmed as sensitive markers of proximal tubular injury and stress that are often altered earlier than creatinine. Previous literature aligns with our results, where the use of KIM-1/NGAL is aligned with current AKI biomarker literature and meta-analyses highlighted their diagnostic utility and pathobiological specificity [40,41].

Our data further confirmed that Cisplatin generates ROS/RNS production, increased lipid peroxidation (MDA), and decreased antioxidant depletion (TAC), together with iNOS induction that amplifies nitrosative damage. Furthermore, the witnessed lessening in MDA and iNOS with elevation of TAC after Secukinumab denoted the mitigation of this axis. Since IL-17A signaling recruits NF-κB and provokes pro-oxidant mediators, its blockade postulates a plausible upstream control point that lessens redox stress and breaks the vicious cycle of inflammation/oxidative injury in the proximal tubule [39,42].

IL-17A is an intense amplifier of stromal and epithelial inflammation; it cooperates with TNF-α/IL-1 to drive NF-κB and p38 MAPK programs, terminating in IL-6 and chemokine production. Our results revealed that Secukinumab reduced phospho-p65 (Ser536) and p38 MAPK in renal tissue, together with lower IL-6 expression, which are concomitant with the role of the Th17/IL-17 axis across kidney diseases and AKI, and also human and preclinical data showing that Secukinumab suppresses IL-17A-dependent NF-κB target cascades. In sepsis models, Secukinumab dampened IκBα/NF-κB activity, and in psoriasis, it quickly regularizes inflammatory transcripts and histology mechanistic outlines that map to the renal milieu here [20,43,44]. At the network level, IL-6 is mutually an outcome and amplifier of NF-κB/STAT3 signaling; restraining IL-17A can consequently indirectly blunt the IL-6 amplifier loop in injured tubules [8,45], which aids in explaining the concordant drops in IL-6 staining and NF-κB activation we witnessed.

Current data indicate that Secukinumab treatment elevates SIRT1 levels compared to the Cisplatin group, providing a mechanistic link to its protective effects against Cisplatin-induced nephrotoxicity. SIRT1 deacetylates the p65 subunit of NF-κB, thereby restraining NF-κB–dependent inflammatory and fibrotic responses in renal tissue exposed to Cisplatin [15,46]. In Cisplatin models, downregulation of SIRT1 permits sustained p65 acetylation, driving chronic inflammation, oxidative stress, and apoptotic signaling. The observed SIRT1 upregulation with Secukinumab likely mitigates these deleterious effects, linking cytokine blockade (IL-17A inhibition) to suppression of NF-κB–mediated inflammatory cascades.

Moreover, Secukinumab treatment elevated AMPKα1 levels, corroborating a cytoprotective metabolic shift. AMPK activation promotes autophagy and inhibits mTOR signaling, which together protects renal cells from Cisplatin-induced oxidative and apoptotic damage [12,47,48]. Collectively, the SIRT1–AMPK axis represents a mechanistic bridge connecting IL-17A neutralization to improved mitochondrial quality, redox homeostasis, and reduced NF-κB–driven inflammation. These findings suggest that targeting inflammatory pathways with Secukinumab enhances intrinsic renal defense mechanisms, providing both anti-inflammatory and anti-apoptotic benefits in the context of Cisplatin-induced AKI [12].

Cisplatin acutely provokes autophagy in tubular cells as an early pro-survival response, but its persistence becomes maladaptive during repair. Beclin-1 is a gatekeeper of the autophagy initiator, while P62/SQSTM1 gathers when autophagic flux is disrupted. Secukinumab treatment in current research led to upregulation of Beclin-1 and concomitant reduction of P62/SQSTM1, indicating enhanced autophagic flux and improved mitochondrial quality control rather than a blockade of autophagy. Mechanistically, this effect is closely linked to inhibition of IL-17A/NF-κB/p38 signaling, which reduces inflammatory and oxidative stress upstream. This is supported by research showing that increasing Beclin-1 activity lessens injury and fibrosis after AKI, comprising Cisplatin models, and is consistent with the canonical Beclin-1/BCL-2 regulatory framework that pairs autophagy to apoptosis. By diminishing inflammatory/oxidative stress upstream (IL-17A → NF-κB/p38). By dampening these stressors, Secukinumab creates a cellular environment that permits autophagy to operate in its adaptive range, supporting tubular survival and promoting orderly repair and these findings are in line with previous studies showing that enhancing Beclin 1-mediated autophagy alleviates injury and fibrosis in Cisplatin-induced AKI [49,50,51] and are supported by the study of Elrashidy et al., 2025, who demonstrated that Secukinumab suppresses IL-17-mediated pyroptosis while enhancing adaptive autophagy [21]. Together, our data provides mechanistic evidence that Secukinumab promotes renal protection by harmonizing autophagy and apoptotic signaling in the context of Cisplatin nephrotoxicity.

The second part of this study also investigated whether Secukinumab, as a selective IL-17A-neutralizing monoclonal antibody, could alter the reaction of human breast cancer MCF-7 cells to Cisplatin, focusing on MTT, Chou–Talalay CI indicating drug-drug interaction, and apoptosis signaling (P53/BAX/ BCL-2). While Secukinumab is not an anticancer drug, the IL-17A axis is distinguished to intensify inflammatory signaling, NF-κB activation, and IL-6 discharge, all of which can boost tumor cell survival and chemoresistance in several tissues. Neutralizing IL-17A, hence has the potential to indirectly trigger cancer cells to cytotoxic agents [52,53,54]. In current data, Cisplatin-treated cells generated a great, concentration-dependent reduction in MCF-7 viability, which is consistent with well-known sources, which showed that Cisplatin activates P53-dependent apoptosis in these cells [52,55]. Furthermore, Secukinumab alone exhibited no intrinsic cytotoxicity, which is accepted as Secukinumab does not interfere with cell proliferation pathways and acts entirely by neutralizing IL-17A [56,57]. Finally, our results revealed that cells treated with Secukinumab and Cisplatin demonstrated further reduction in viability than Cisplatin alone. While Secukinumab does not destroy cells directly, IL-17A block revealed suppression of NF-κB-mediated inflammatory signaling in numerous cell systems. This drop in survival- indorsing inflammatory pathways that may drop the apoptotic threshold and permit Cisplatin to be more efficient [58,59]. Our data suggested that Secukinumab modulated the cellular environment that enhanced Cisplatin’s action, despite having no direct cytotoxic activity.

The Chou–Talalay model is the gold-standard quantitative system for defining true drug synergy using dose-effect relationships [36]. Using the Chou–Talalay method, the combination of Secukinumab + Cisplatin produced CI < 1 at many concentration pairs, indicating synergism. Mechanistically, synergy here is reasonable as neutralizing IL-17A diminished NF-κB activation, which is a known pro-survival and pro-resistance pathway in various cancers. By eradicating inflammatory reinforcement, Secukinumab likely makes MCF-7 cells more vulnerable to DNA damage- triggered apoptosis [58]. Our observed synergistic interaction suggested that Secukinumab potentiated Cisplatin-induced cytotoxicity, potentially allowing dose reduction of Cisplatin while maintaining or enhancing its anticancer efficacy.

Apoptotic Signaling (P53, BAX, BCL-2) is the conventional apoptotic pattern for Cisplatin-treated MCF-7 cells [39,58,60]. Our results revealed that Cisplatin alone upregulated P53 and BAX and downregulated BCL-2. These changes reflect a pronounced shift toward apoptosis, as P53 is a key tumor suppressor that responds to DNA damage by transcriptionally activating pro-apoptotic genes, including BAX, while repressing anti-apoptotic proteins such as BCL-2 [61,62]. Secukinumab alone did not induce a significant change in P53/BAX/BCL-2. Since Secukinumab does not directly act on apoptotic machinery. Secukinumab + Cisplatin combination enhanced apoptotic signaling: upregulated P53/BAX expression and stronger downregulation of BCL-2. This amplified apoptotic profile supports the CI findings and indicates that blocking IL-17A removes survival signaling, allowing Cisplatin to push the cell more decisively toward apoptosis. Thus, observed upregulation of P53 suggests enhanced DNA damage signaling induced by Cisplatin, which is further potentiated in the presence of Secukinumab.

BAX is a critical pro-apoptotic member of the BCl-2 family that promotes mitochondrial outer membrane permeabilization, leading to cytochrome c release and caspase activation, hallmark events of intrinsic apoptosis [63,64]. Therefore, increased BAX levels indicate activation of the intrinsic (mitochondrial) apoptotic pathway. In contrast, BCL-2 functions to preserve mitochondrial integrity and inhibit apoptosis; thus, its downregulation removes a key survival signal and facilitates apoptotic progression [65]. Importantly, the ratio between BAX and BCL-2 is considered a decisive determinant of cell fate, with higher ratios correlating with increased apoptotic susceptibility [66]. The significant increase in the BAX/BCL-2 ratio observed in the combination group indicates a strong commitment toward apoptosis rather than survival.

Mechanistically, while Cisplatin directly induces DNA damage and activates the P53-dependent apoptotic pathway [67], Secukinumab appears to enhance this effect indirectly through modulation of inflammatory signaling. Neutralization of IL-17A likely suppresses NF-κB activation, a pathway known to promote cell survival and upregulate BCL-2 expression [68,69]. Inhibition of this pro-survival signaling may sensitize cancer cells to Cisplatin-induced apoptosis, thereby amplifying P53 activation and shifting the balance toward pro-apoptotic signaling.

Secukinumab likely defends the kidney *via* an anti-cytokine strategy: IL-17A neutralization/reduced NF-κB/activation, P38 MAPK/reduced IL-6 production in renal parenchyma. Lower iNOS/ROS burden (↓MDA and ↑TAC), mitigating mitochondrial and membrane injury. Re-engagement of SIRT1–AMPK homeostasis, which inhibits acetyl p65 and pro-inflammatory transcription, promotes advantageous bioenergetics and facilitates adaptive autophagy. Autophagy tuning (Beclin-1↑ and P62/SQSTM1↓) suggests improved proteostasis and mitophagy, reduced apoptotic priming, and better structural recovery. This cascade reconciles the biochemical, molecular, IHC, and histology readouts into a single mechanistic narrative consistent with modern Cisplatin-AKI biology.

In MCF-7, the combined data support the following mechanism: Cisplatin induces DNA damage that activates P53/BAX and reduces BCL-2, enhancing apoptosis. Although Secukinumab has no inherent anticancer activity, it significantly enhanced the apoptotic and cytotoxic effects of Cisplatin in MCF-7 cells by reducing IL-17A–driven NF-κB inflammation, thus increasing P53 and BAX activation, suppressing BCL-2, and inducing concentration-dependent synergism in CI analysis. These findings highlight the significance of the cytokine-inflammatory axis in tumor cell survival and support the concept that immunomodulatory biologics may improve chemotherapy response even without directly targeting malignant cells.

Limitation of the study: This study acknowledges that future investigations will employ flow cytometry to further delineate the specific mode of cell death induced by this combination. Additionally, while MCF-7 provided valuable initial insights, future studies should include triple-negative breast cancer (TNBC) models (like MDA-MB-231) to fully validate the synergistic potential of Secukinumab in a clinical context where Cisplatin is the first-line treatment. In the present study, the focus was directed toward IL-17–mediated immune mechanisms; therefore, an antioxidant comparator was not included. Future studies may explore potential synergistic effects between antioxidants and immunomodulatory approaches.

## 5. Conclusions

Our findings demonstrate that Secukinumab robustly protects against Cisplatin-induced acute kidney injury, Secukinumab attenuated oxidative stress and inflammatory signaling while promoting autophagy, resulting in preserved renal histology and function. Simultaneously, Secukinumab has no direct cytotoxicity, but potentiates Cisplatin effects indirectly *via* IL-17A/NF-κB inhibition. These dual reno-protective and antitumor effects highlight the therapeutic potential of Secukinumab in combination with Cisplatin.

## Figures and Tables

**Table 1:** Renal histopathological scores [34].

Score	Tubular Damage	Tubular Dilation and Cast	Inflammation	Fibrosis
0	None	None	None	None
1	Minimal, few	Minimal dilation with rare to few intraluminal cast	Few, rare	Few
2	Mild to moderate tubular degeneration	Minimal to mild dilation with moderate number of intraluminal cast	Mild, focal	Moderate interstitial fibrosis
3	Diffuse, many tubular necroses	numerous intraluminal cast	Moderate to severe coalescing interstitial inflammatory aggregates	Severe dense, interstitial fibrosis

**Table 2:** The impact of Secukinumab (10 & 20 mg/kg) on Cisplatin-induced alterations in serum kidney function biomarkers and LDH.

	Control Group	Cisplatin Group	Secukinumab Control Group	Secukinumab (10 mg/kg) Group + Cisplatin	Secukinumab (20 mg/kg) Group + Cisplatin
Serum Creatinine (mg/dL)	0.97 ± 0.15	2.75 ± 0.33	0.96 ± 0.15 ^##^	2.15 ± 0.39 ^##^*	1.81 ± 0.32 ^##^**
BUN (mg/dL)	17.26 ± 1.68	73.32 ± 5.01	16.30 ± 2.65 ^##^	70. 82 ± 7.54 ^##^	57.81 ± 9.30 ^##^*
LDH (U/L)	981.60 ± 177.48	1921.40 ± 522.90	870.80 ± 180.17 ^##^	1483.80 ± 110.82	1354.00 ± 197.75 *

Data are presented as mean ± SD (*n* = 5). Statistical evaluation was carried out using one-way ANOVA, followed by the Tukey–Kramer post hoc test. ^##^ indicates a highly significant difference compared with the control group, *, ** significant and highly significant differences matched to the Cisplatin group, respectively. BUN: Blood Urea Nitrogen; LDH: Lactate Dehydrogenase.

**Table 3:** Combination index (CI) values for Cisplatin and Secukinumab combination for breast cancer cell line (MCF-7) (Comparison of IC_50_ values of Cisplatin alone and in combination with Secukinumab).

Cisplatin (µM)	% Cell Inhibition	CI Value	Interaction
0.5	19%	0.88	Synergistic
1	33%	0.79	Synergistic
2	45%	0.7	Strong Synergistic
5	55%	0.62	Strong Synergistic
10	72%	0.55	Strong Synergistic
20	88%	0.48	Very strong Synergistic

CI: combination index, CI < 1 synergistic effect, CI > 1 antagonistic association.

## Data Availability

The datasets generated during and/or investigated through the existing analysis are accessible from the corresponding author on reasonable request.

## Abbreviations

AMPKα1: AMP-activated protein kinase α1; ANOVA: Analysis of Variance; BUN: Blood urea nitrogen; CI: Combination index; DMEM: Dulbecco’s Modified Eagle Medium; DMSO: Dimethyl sulfoxide; H&E: Hematoxylin and eosin; IC_50_: Half-Maximal Inhibitory Concentration; IL-6: Interleukin-6; iNOS: Inducible nitric oxide synthase; IP: Intraperitoneal; KIM-1: Kidney injury molecule 1; LDH: Lactate dehydrogenase; MCF-7: Michigan Cancer Foundation-7; MDA: Malondialdehyde; NF-κB p65: Nuclear factor kappa B; NGAL: Neutrophil gelatinase-associated lipocalin; P38 MAPK: P38 mitogen-activated protein kinase; P62/SQSTM1: Sequestosome-1; PBS: Phosphate-buffered saline; SC: Subcutaneously; SD: Standard deviation; SIRT1: Nad-dependent protein deacetylase sirtuin-1; TAC: Total antioxidant capacity.
